# Argonaute-associated short introns are a novel class of gene regulators

**DOI:** 10.1038/ncomms11538

**Published:** 2016-05-13

**Authors:** Thomas B. Hansen, Morten T. Venø, Trine I. Jensen, Anne Schaefer, Christian K. Damgaard, Jørgen Kjems

**Affiliations:** 1 Department of Molecular Biology and Genetics (MBG), Interdisciplinary Nanoscience Center (iNANO), Aarhus University, C.F. Moellers Alle 3, Build 1130, Aarhus 8000, Denmark; 2 Friedman Brain Institute, Icahn School of Medicine at Mount Sinai, 1425 Madison Avenue, New York, New York 10029, USA

## Abstract

MicroRNAs (miRNAs) are short (∼22 nucleotides) regulators of gene expression acting by direct base pairing to 3′-UTR target sites in messenger RNAs. Mature miRNAs are produced by two sequential endonucleolytic cleavages facilitated by Drosha in the nucleus and Dicer in the cytoplasm. A subclass of miRNAs, termed mirtrons, derives from short introns and enters the miRNA biogenesis pathway as Dicer substrates. Here we uncover a third biogenesis strategy that, similar to mirtron biogenesis, initiates from short introns but bypasses Dicer cleavage. These short introns (80–100 nucleotides), coined agotrons, are associated with and stabilized by Argonaute (Ago) proteins in the cytoplasm. Some agotrons are completely conserved in mammalian species, suggesting that they are functionally important. Furthermore, we demonstrate that the agotrons are capable of repressing mRNAs with seed-matching target sequences in the 3′-UTR. These data provide evidence for a novel RNA regulator of gene expression, which bypasses the canonical miRNA biogenesis machinery.

## Abstract (web summary)

MicroRNAs are important small regulatory RNAs produced by sequential Drosha and Dicer cleavage. Here the authors describe 'agotrons', a subclass of small RNAs that bypass the canonical biogenesis machinery.

## Graphical abstract

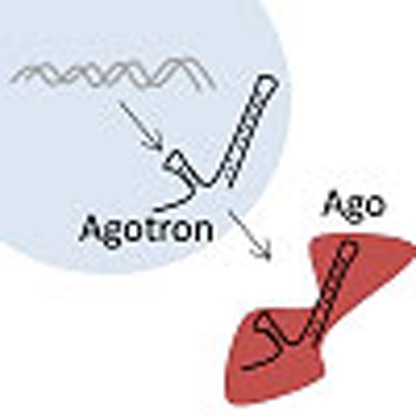

MicroRNAs (miRNAs) are the ∼22 nucleotide products of a two-step enzymatic maturation process facilitated by Drosha and Dicer, respectively^1^. After biogenesis, the miRNA is then incorporated into the RNA-induced silencing complex (RISC) and directs the binding to specific regions in the 3′-untranslated region (3′-UTR) of mRNAs by Watson–Crick base pairing between the miRNA seed (position 2–8) and the target^2^. This promotes translational repression and ensuing mRNA destabilization by deadenylation and decapping of the targeted transcript^3^. A subset of miRNAs, so-called mirtrons, bypass the initial Drosha cleavage and instead depend on splicing and debranching to produce the pre-miRNA Dicer substrate^4^,^5^,^6^. On Dicer cleavage the mirtrons enter the canonical miRNA pathway and are therefore functionally identical to the Drosha-dependent miRNA species. To date, one miRNA, miR-451, has been shown to be generated in a Dicer-independent manner. The endonucleolytic slicer activity of Ago2 cleaves the pre-miR-451 hairpin into a ∼30 nt RNA, which is trimmed into a conventional ∼22 nt miRNA-like species by 3′-exonucleolytic activity^7^,^8^.

Here we describe a novel class of non-coding RNA species derived from introns, which we coin ‘agotrons'. Unlike mirtrons, agotrons are not cleaved by Dicer and the mature form resembles the pre-miRNA. Agotrons are highly conserved in mammals and are widely associated and stabilized by Argonaute (Ago) proteins. Functionally, agotrons induce target repression in an miRNA-like manner and we identify endogenous targets for the highly conserved agotron in the last intron of the *Pkd1* gene.

## Results

### Ago2 HITS-CLIP

**Figure 1:**
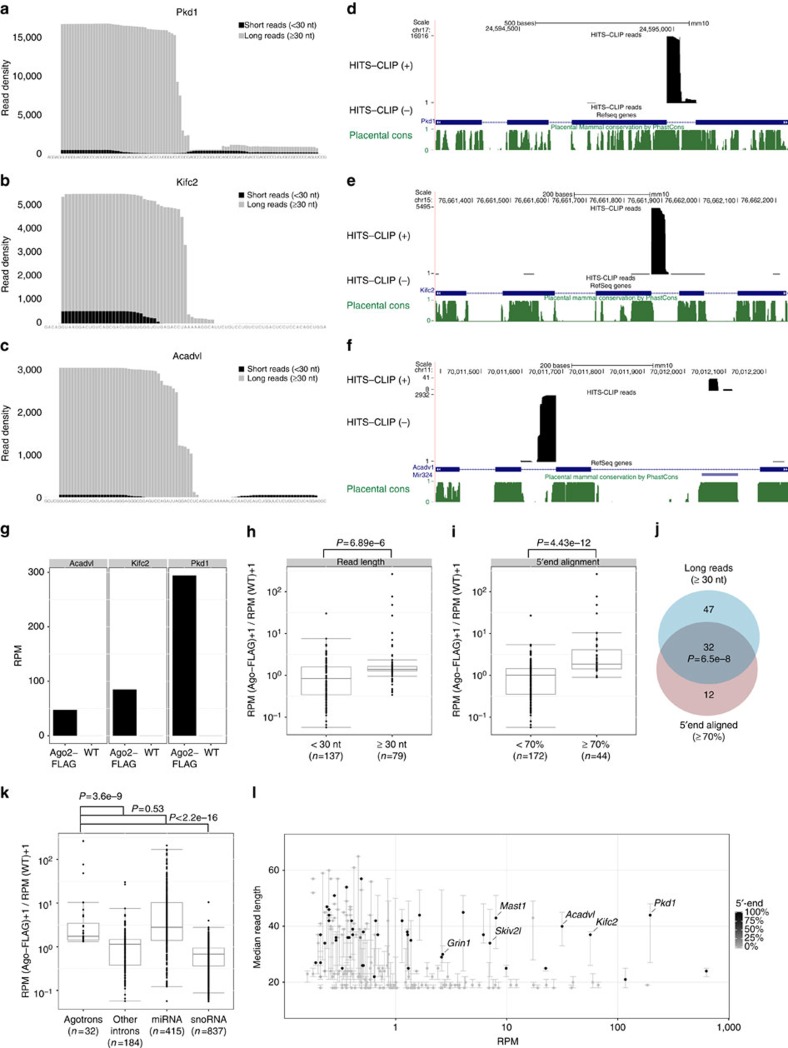
Ago2 HITS-CLIP analysis of short introns. (**a**–**c**) Based on Ago2 HITS-CLIP (Tan *et al*.^9^); stacked density plots of putative agotron, *Pkd1* (chr17:24594977-24595071(+), mm10) (**a**), *Kifc2* (chr15:76661872-76661945(+), mm10) (**b**) and *Acadvl* (chr11:70011598-70011683(−), mm10) (**c**), respectively, with reads divided into long (≥30 nt, grey) or short (<30 nt, black). UCSC screendump of selected introns, *Pkd1* (**d**), *Kifc2* (**e**) and *Acadvl* (**f**) including the flanking regions, with HITS-CLIP read density (black) where (+) and (−) reflect the read direction, exon–intron annotation (blue) and placental phastcons conservation score profile (green). (**g**) Number of reads mapping to selected agotrons, *Acadvl*, *Kifc2* and *Pkd1* (as indicated), from Ago2 HITS-CLIP with Flag-tagged Ago2 expression (Ago2-FLAG) or WT. Boxplot of FLAG-Ago enrichment calculated as log2 (RPM in FLAG-Ago HITS-CLIP/RPM in WT HITS-CLIP) of intronic region with median read length ≥30 nt or <30 nt (**h**) or with fraction of 5′-end aligned reads ≥70% or <70% (**i**). *P*-values are calculated using Wilcoxon rank-sum test. (**j**) Venn diagram depicting the intersection between introns with long read length (*n*=79) and high 5′-end alignment (*n*=44) out of all the expressed introns (read count ≥20, *n*=216). *P*-value is calculated using *χ*^2^-test. (**k**) Boxplot of FLAG-Ago enrichment (as in **h** and **i**) of agotron, other introns, miRNAs and small nucleolar RNAs (snoRNAs). *P*-values are calculated using Wilcoxon rank-sum test. (**l**) All short annotated introns between 40 and 150 nucleotides (from UCSC gene annotation, mm10) are plotted as median read length by read count. Only introns with at least 20 counts are shown. The points are colour scaled according to the percentage of reads aligning to the 5′-end of the intron. Error bars reflect 5 and 95 percentiles.

While analysing the Ago2-CLIP data set from mouse forebrain neurons^9^, we noticed a high density of long reads exceeding 30 nucleotides (nt) in length across short introns in *Pkd1*, *Acadvl* and *Kifc2* genes (Fig. 1a–c). This is in contrast to conventional mirtrons and miRNAs where long reads were completely absent (Supplementary Fig. 1a,b) and where median read length for all expressed miRNA and mirtrons as expected were ∼22 nt (Supplementary Fig. 1c). The accumulation of reads was highly intron specific, as neighbouring introns in the respective genes were devoid of reads (Fig. 1d–f). High-throughput sequencing of RNAs isolated by cross-linking immunoprecipitation (HITS-CLIP) was performed with anti-FLAG antibody from the forebrain of mice with a neuron-specific FLAG-Ago2 expression or their wild-type (WT) littermate controls. When comparing reads from FLAG-Ago2 and WT data sets, it is evident that the observed introns were indeed FLAG-Ago2 specific (Fig. 1g). We analysed all the reads mapping onto annotated murine introns between 40 and 150 nucleotides to potentially recapitulate this phenomenon genome wide. We found 216 introns with at least 20 reads out of which 79 had a long median read length. Interestingly, the introns with long median read length were significantly enriched in Ago2-FLAG compared with the remaining introns with shorter reads (Fig. 1h and Supplementary Fig. 1d). Apart from long read length, the Ago2-associated intronic reads aligned predominantly to the 5′-end of the intron (Fig. 1i and Supplementary Fig. 1e), allowing a single-nucleotide offset in line with the decapitation recently observed in mirtrons^10^. In fact, long read length and high 5′-end homogeneity seem to be significantly coupled features (Fig. 1j), suggesting that these introns represent non-random biological RNA species. Hence, we propose that a subclass of Ago2-associated introns, coined ‘agotrons', can be defined by long and 5′-end aligned reads in Ago2 HITS-CLIP data sets. Similar to miRNAs, these distinct features are highly enriched in FLAG-Ago2 reads compared with other introns and small nucleolar RNAs (Fig. 1k and Supplementary Fig. 1h,i). Moreover, only 3 of the 32 agotron-like species are annotated in mirbase as mirtrons and agotrons in general exhibited a higher Ago2-FLAG enrichment compared with other introns with 5′-end aligned short reads annotated mostly as mirtrons (Supplementary Fig. 1f). Collectively, abundant agotrons are distinguishable from other intronic regions by exhibiting long read length and a high fraction of 5′-end aligned reads in the Ago2 HITS-CLIP data (Fig. 1j). Using these characteristics, we scrutinized other HITS-CLIP data sets, to validate these Ago2-associated introns. Based on HITS-CLIP data from Darnell's lab^11^, which is performed using an Ago2 antibody against the endogenous Ago2 protein from total embryonic mouse brain, we again observed the presence of agotron-like short introns (Supplementary Fig. 2a). In fact, a noteworthy overlap between the highly abundant introns with 5′-aligned long reads was observed between the two data set (for example, *Acadvl*, *Grin1* and *Mast1*). In the HITS-CLIP procedure, the Ago2–RNA complex weighs 110 or 130 kDa depending on the length of the Ago2-associated RNA^11^. The agotrons were highly enriched in the 130-kDa fraction compared with miRNAs that predominantly accumulate in the 110-kDa fraction (Supplementary Fig. 2b–d), emphasizing that these species are indeed longer than conventional miRNAs. Similar analyses on other murine Ago2 HITS-CLIP experiments using endogenous Ago2 immunoprecipitation (IP)^12^,^13^ also revealed the presence of agotron-like species (Supplementary Fig. 3a,b), demonstrating that these are not artefacts of FLAG-Ago2 expression. Two recurring agotrons, *Pkd1* and *Mast1*, showed a remarkable cross-species conservation (Supplementary Fig. 4a,b) and a peculiar secondary structure predicted by LocARNA-P^14^ (Supplementary Fig. 4c,d). In fact, the last intron of the *Pkd1* gene, which is the agotron-producing intron, has been characterized as more conserved than the protein-coding region of the host gene^15^. Therefore, we turned to human Ago HITS-CLIP data sets^16^,^17^,^18^,^19^,^20^ based on either pan-Ago IP or Ago2-specific IP, to disclose agotron-like species further (see Supplementary Data 1). Here, *PKD1* and *MAST1* were once again classified as agotrons in most data sets (Supplementary Fig. 3c–g). In case of data sets lacking *PKD1* or *MAST1* validation, this was due to lack of expression and not lack of agotron features. In addition, other reoccurring human-specific and non-conserved agotrons, for example, *IVD* and *SLC4A2* emerged (Supplementary Fig. 3c–g).

### Agotrons are stabilized by Ago proteins

**Figure 2:**
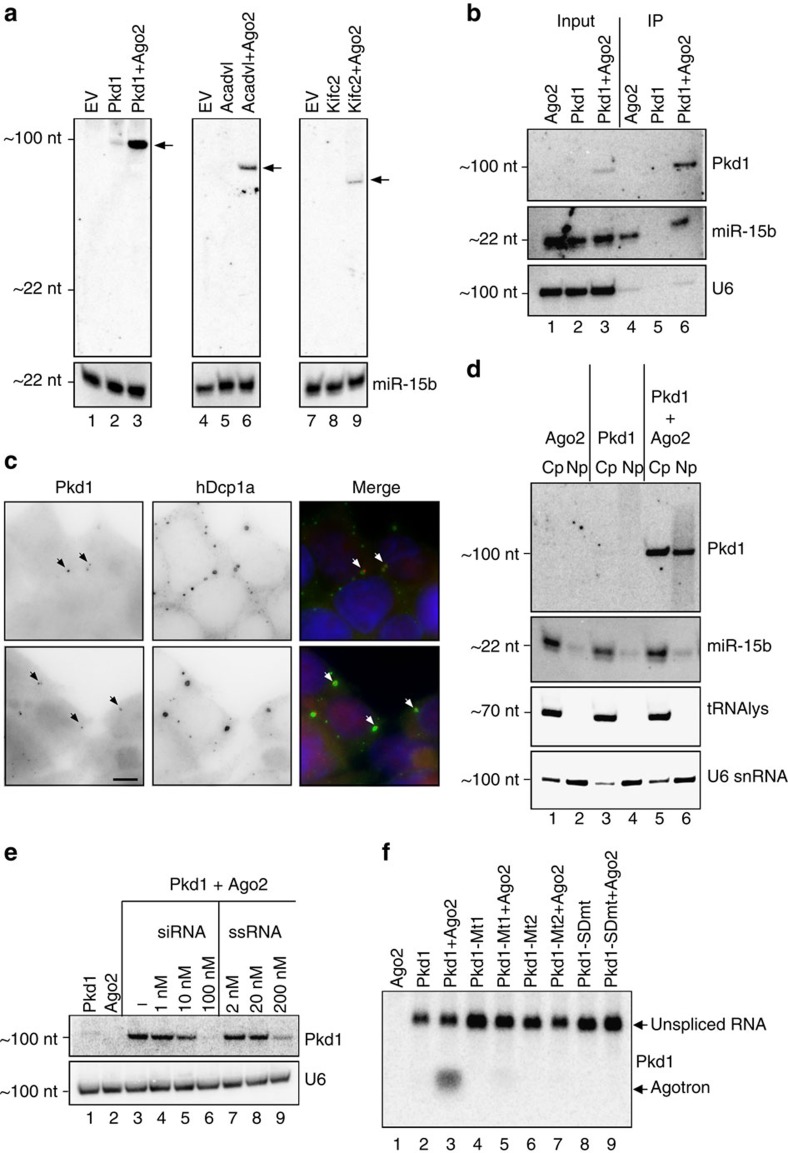
Agotrons are stabilized by Ago2 expression. (**a**) Northern blotting of RNA from HEK293 cells transiently expressing either an empty vector (EV, lane 1, 4 and 7), agotron minigene (*Pkd1*, *Acadvl* or *Kifc2*; lane 2, 5 and 8) or co-expressing Ago2 with agotron minigene (lane 3, 6 and 9). Northern membranes were individually probed for the expressed agotron using 22-nt-long oligos complementary to the 5′-end of the respective agotrons. Arrows indicate bands corresponding to agotrons. miR-15b serves as loading control. (**b**) PAGE northern using RNA from input and IP fractions from HEK293 cells expressing *Pkd1* agotron, Ago2 or both as denoted. Membranes were probed for *Pkd1* agotron, miR-15b (positive control) and U6 (negative control). (**c**) Fluorescence *in-situ* hybridization/immunofluorescence (FISH/IF) on HEK293 cells transiently overexpressing Ago2 and *Pkd1*-agotron using cy5-labelled probes against *Pkd1*- and hDcp1A-specific antibody as a processing body (PB) marker (upper and lower panels). Arrows point to examples of co-localized *Pkd1* and PB. Scale bar, 5 μm. (**d**) PAGE northern showing subcellular fractionation of HEK293 cells expressing Ago2, *Pkd1* or both. Membrane was probed as indicated. U6 and tRNA-lys represent nuclear and cytoplasmic markers, respectively. Cp, cytoplasm; Np, nucleoplasm. (**e**) Competition assay with increasing amount of double-stranded small interfering RNA (siRNA) or single-stranded RNA (ssRNA) as indicated. U6 serves as loading control. (**f**) Agarose northern with *Pkd1* mutants (Mt1 and Mt2) and a splice donor (SD) mutant using a probe against the unmodified agotron 3′-end. Top band corresponds to unspliced RNA derived from minigene expression and the lower band corresponds to mature agotrons, as indicated to the right.

To validate the HITS-CLIP data, we selected three murine agotrons: *Pkd1*, *Acadvl* and *Kifc2*. We constructed minigene vectors comprising the agotron-containing intron and the immediate flanking exons (see Supplementary Fig. 5a). The constructed minigenes were then transfected with or without Ago2 co-transfection into HEK293 cells. All three agotrons produced a distinct product migrating according to linear, full-length intron sizes (94, 85 and 73 nt, respectively, Fig. 2a); however, the agotrons were almost solely detectable on Ago2 co-expression, suggesting that without Ago2, the agotrons are either inefficiently spliced or inherently unstable. To test the former hypothesis, we assayed the relative levels of spliced minigene in the presence or absence of Ago2 co-expression by reverse transcriptase PCR (RT–PCR) (Supplementary Fig. 5b). Here, the ratio between spliced and unspliced were unchanged irrespective of Ago2 expression, suggesting that Ago2 has a stabilizing effect on the agotrons, which also is in agreement with the high Ago2–agotron association seen in various HITS-CLIP data. For all of the agotrons analysed, we failed to detect RNA fragments in the mature miRNA size range. In subsequent analyses, we focused on *Pkd1* as a highly abundant and conserved representative of agotrons. First, we performed Ago2 IP to validate the interaction between Ago2 and *Pkd1* (Fig. 2b). Here, the *Pkd1*-derived agotron was efficiently immunoprecipitated similar to the endogenously expressed miR-15b, whereas U6 was not enriched in Ago2 IP fractions. Furthermore, Ago2 only associates with the excised intron and not the unspliced transcript (Supplementary Fig. 5c), suggesting that Ago2 prefers an accessible 5′-end in agreement with Ago crystal structures showing a binding pocket for 5′-ends at the interface between the PIWI and MID domain^21^. To determine whether the observed interaction was Ago2 specific, we co-transfected the *Pkd1* agotron with Ago1 expression plasmid. Here, similar to Ago2, stabilization of *Pkd1* agotron is observed, suggesting that at least this agotron has no Ago1/2 preference (Supplementary Fig. 5d). Based on combined fluorescence *in-situ* hybridization/immunofluorescence using five tiled cy5-labelled probes for the *Pkd1*-derived agotron and antibodies against the processing body marker hDcp1a^22^, we observed a clear cytoplasmic co-localization between the agotron and processing bodies (Fig. 2c), which is unusual for intronic RNAs, but known to be enriched in Ago-loaded complexes^23^. Without Ago2 or *Pkd1* overexpression, no agotron signal was observed. Cellular fractionation also revealed an enrichment of *Pkd1* agotron in the cytoplasm (Fig. 2d). However, surprisingly, the agotron was also clearly detectable in the nuclear fraction, but only on Ago2 co-expression, suggesting either that the Ago2–agotron complex is imported after association, or that Ago2 interacts and stabilizes the agotron already in the nucleus. Finally, the stabilizing effect of Ago2 was abolished in a concentration-dependent manner on co-transfection with small interfering RNA and, to a lesser extent, with single-stranded RNA (Fig. 2e), indicating, as expected, that agotrons and small interfering RNA compete for the same binding domain on Ago2.

To elucidate the sequence requirement for the Ago2 interaction, we constructed a series of *Pkd1* mutants (Supplementary Fig. 5e). As it has been proposed that Ago2 has a preference for G-rich regions^12^, we mutated a stretch of G's (Mt1) to determine whether this was important for Ago2 association. Here, no agotron production was observed (Fig. 2f); however, the introduced mutation disrupted splicing and the Ago2 association was therefore unresolved. Another mutation (Mt2) disrupts the distal stem without changing the nucleotide composition. Here, the mutation had no noticeable effect on splicing, but no agotron production was observed, suggesting that distal stem is required for agotrons to become associated with and stabilized by Ago2. However, a more subtle mutation in the distal stem (Mt3) exhibited agotron production similar to WT *Pkd1* (Supplementary Fig. 5f). Finally, when mutating the splice donor (GU->CU), splicing is unproductive and consequently no agotron is observed as expected (Fig. 2f). Collectively, for agotrons to be associated with and stabilized by Ago2, specific structural requirements seem important that are yet to be thoroughly and systematically disclosed.

### Agotrons repress target genes in a miRNA-like manner

**Figure 3:**
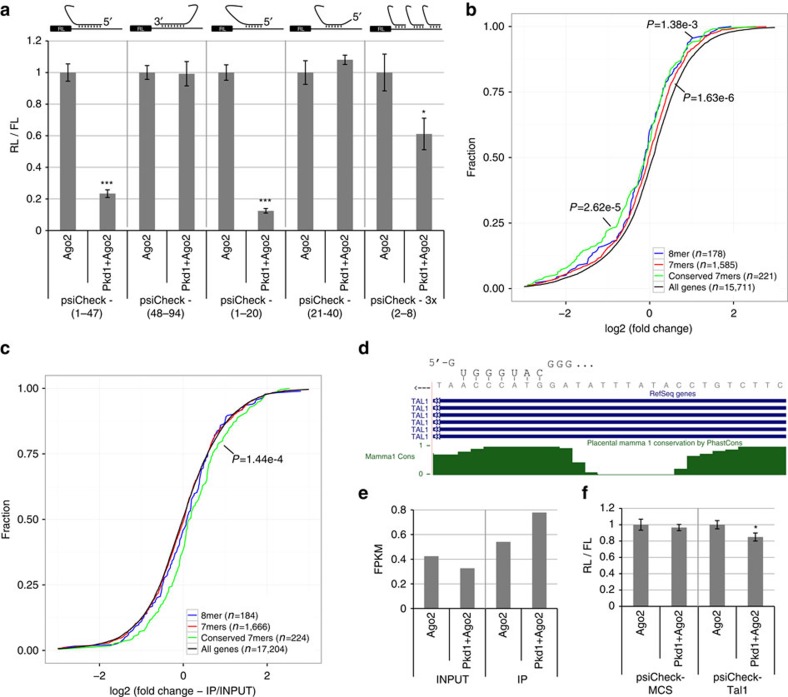
Agotrons are functional target repressors. (**a**) Luciferase reporter assays using psiCheck2 vectors with either a perfect target site for the indicated *Pkd1* subregions: 1–47, 48–94, 1–20, 21–40 or 3x8mer seed matches (3x(2–8)) inserted in *Renilla* (RL) 3′-UTR as schematically depicted. The luciferase reporters were co-expressed with either Ago2 alone (Ago2) or together with *Pkd1* (*Pkd1*+Ago2) and relative luminescence was determined by the *Renilla:Firefly* (RL/FL) ratio. Values were normalized to Ago2 co-expression. (**b**) Cumulative plot of log2 (fold change) from RNAseq quantification of RNA with putative targets in Ago2- or Ago2+*Pkd1*-expressing HEK293 cells. Transcripts with either a putative 8mer, 7mers (7mer-m8 and 7mer-1A) and conserved 7mers (phastcons 46way placental conservation score above 50%) target sites for *Pkd1* are plotted. *P*-values are obtained by Wilcoxon rank-sum test. (**c**) Cumulative plot of log2 (fold change) from RIPseq of Ago2 IP. Fold changes are calculated as Ago2+*Pkd1* (IP/INPUT)/Ago2 (IP/INPUT). *P*-values are obtained by Wilcoxon rank-sum test. (**d**) UCSC screenshot of putative *Pkd1* target site in the 3′-UTR of *TAL1* (chr1:47684934-47684961, hg19, *TAL1* is on the negative strand) with predicted seed match interaction. The placental phastcons conservation score profile is depicted in green. (**e**) *TAL1* expression (in FPKM) determined by RNAseq from input and IP samples expressing Ago2 with or without co-expression of *Pkd1*. (**f**) As in **a**, using psiCheck vectors with multiple cloning site (MCS) or with a inserted region of the *TAL1* 3′-UTR. Error bars represent s.d. (*n*=3). *P<0.05; ***P<0.001, two-tailed *T*-test.

To assess whether agotrons are capable of target gene suppression in a miRNA-like manner, we constructed luciferase reporter constructs (psiCheck vectors) harbouring in the 3′-UTR a stretch of perfect complementary nucleotides towards either the 5′-part (1–47) or the 3′-part (48–94) of the *Pkd1* agotron. Here, co-expressing the psiCheck vectors with Ago2 and *Pkd1* showed a potent suppression of the 5′-part complementary reporter, but not the 3′-part reporter (Fig. 3a). Dissecting the target preference further revealed that only reporters containing the complementary sequence of the first 20 nt (1–20) of the 94 nt agotron were susceptible for suppression, whereas the expression of constructs containing the downstream 20 nt (21–40) was not affected by *Pkd1* agotron expression (Fig. 3a). This was also observed for the *Acadvl* agotron (Supplementary Fig. 6a). In fact, the *Pkd1* agotron was able to suppress a reporter harbouring three consecutive miRNA-like 8mer seed matches (3 × (2–8), Fig. 3a). This suggests that agotrons discriminate targets from non-targets similar to conventional miRNAs, governed predominantly by seed matches. To construct a *Pkd1* mutant with a modified seed, we introduced a TA>AT mutation into the region encoding the non-basepaired loop of the 5′-part of *Pkd1* (Supplementary Fig. 6b) and designed a corresponding luciferase seed reporter. Unfortunately, this Pkd1 mutant was not expressed, potentially due to interference with splicing or Ago2 binding (Supplementary Fig. 6c); however, expression of WT *Pkd1* was unable to confer repression of the luciferase seed reporter (Supplementary Fig. 6d).

To identify potential endogenous targets, we expressed Ago2 with or without co-expression of the *Pkd1* agotron in HEK293 cells and performed RNAseq on total RNA. Here, a significant decrease of genes harbouring a putative 8mer or 7mers (7mer-m8 or 7mer-1A) in the 3′-UTR was observed (Fig. 3b). The most effectively suppressed class of genes was the conserved subset of 7mers (Fig. 3b, green), corroborating the high cross-species conservation of *Pkd1*. We also sequenced the Ago2 IP fractions with or without *Pkd1* co-expression, to determine whether putative targets are enriched. Importantly, the RNAs harbouring conserved *Pkd1* target sites displayed a clear enrichment, while a general selective enrichment of all putative target genes was not significant (Fig. 3c). Based on ‘sliding seed' windows, the *Pkd1* agotron has a clear effect on targets harbouring 7-nt matches with flanking A towards the 5′-end region of the agotron (Seeds: 2-8A, 3-9A and 4-10A; Supplementary Fig. 7a,b). The strong deregulation and Ago2 association of 3-9A seed targets possibly reflect that the flanking A opposes the U in position 2 of the agotrons. Therefore, we also performed 8-nt complementary matches but with no constrain on the flanking nucleotide (Supplementary Fig. 7c,d). This showed a clear preference for two to nine seed matches and suggests that an extended seed, at least for *Pkd1*, is favoured.

One selected target gene candidate with a conserved 8mer site in the 3′-UTR, *TAL1* (Fig. 3d), was selectively downregulated in total RNA and co-immunoprecipitated in *Pkd1*-expressing cells (Fig. 3e). Testing the putative 8mer in a luciferase reporter assay revealed a modest but significant repression of relative luminescence (Fig. 3f). Collectively, this suggests that agotrons are a new class of functional Ago-associated non-coding RNAs with miRNA-like capabilities.

### Agotrons are Dicer independent

**Figure 4:**
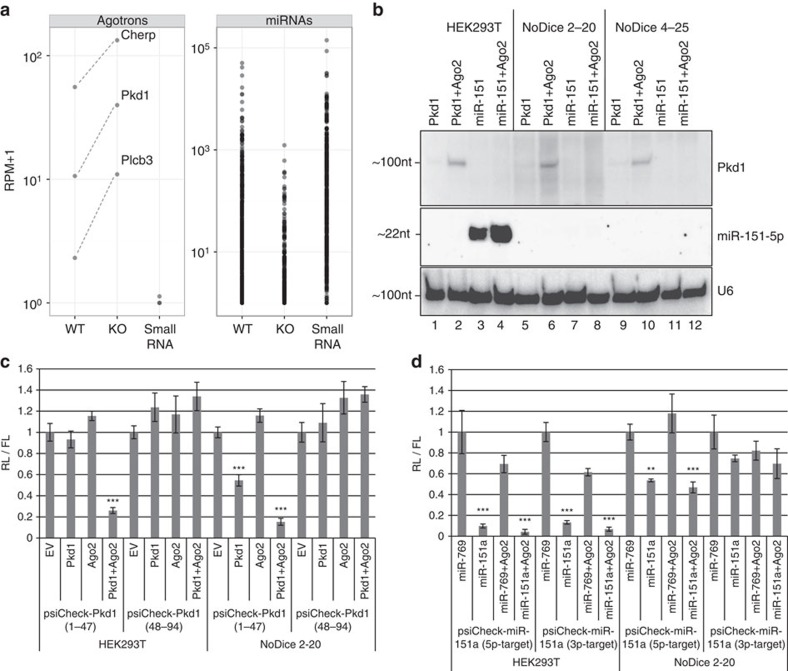
Agotrons are Dicer independent. (**a**) RPM on agotrons or miRNAs obtained from Ago2 HITS-CLIP performed on WT or Dicer-null murine embryonic stem cells (mESCs) (KO) or from total small RNA sequencing (small RNA). Data set is from Leung *et al*.^12^ (**b**) Expression of *Pkd1* or miR-151 with or without Ago2 coexpression in WT HEK293T cells or two Dicer-null HEK293T cells, NoDice 2–20 and NoDice 4–25 (see Methods for details). (**c**,**d**) Luciferase reporter assays performed in either WT (HEK293T) or Dicer KO (NoDice 2-20) HEK293T cells. Reporter constructs for *Pkd1*, psiCheck (1-47) and psiCheck (48-94) (as in Fig. 3a) (**c**) or for the 5*p* and 3*p* arm of miR-151a (**d**) were coexpressed with *Pkd1* or miR-151a expression vector and Ago2 as indicated. Relative luminescence was monitored and normalized. Error bars represent s.d. (*n*=3). **P<0.01; ***P<0.001, two-tailed *T*-test against EV/miR-769 co-expression.

Based on HITS-CLIP and northern blot analyses, agotrons exist predominantly as full-length introns. However, if processed into small RNA by Dicer, this would indiscriminate agotrons from conventional mirtrons and the observed suppressive effect on target genes could be facilitated by an inefficiently produced and undetectable fraction of miRNA-sized agotrons. To test whether Dicer is required for the observed agotron-mediated knockdown effect, we initially turned to the Ago2 HITS-CLIP data set from WT and Dicer knockout (KO) murine embryonic stem cells^12^. Only three introns were here classified as agotrons (Supplementary Fig. 3b), but in all three cases the Ago2 association was enriched in Dicer KO cells, whereas miRNA reads in general are depleted in Dicer KO (Fig. 4a). Furthermore, small RNA sequencing of WT murine embryonic stem cell showed practically no agotron-derived reads compared with HITS-CLIP, whereas miRNA counts in WT HITS-CLIP and small RNA sequencing were comparable (Fig. 4a).

To further disclose the role of Dicer in agotron biogenesis and function, we turned to Dicer-null HEK293T cells (HEK293T NoDice 2-20 and HEK293 NoDice 4-25) that have been previously established^24^. Here, the expression of *Pkd1* agotron was unaffected (Fig. 4b), whereas the biogenesis of a transiently expressed miR-151 was completely abolished (Fig. 4b). Measuring the knockdown potential of agotrons in the Dicer-null background, the agotron remained functional on a 5′-complementary target site and incapable of targeting 3′-complementary sites (Fig. 4c) similar to effects observed in the parental HEK293T cells (Fig. 4c). In fact, the suppressive effect of the *Pkd1* agotron increased slightly in the Dicer-null background, probably due to reduced competition for Ago2 binding.

This strongly suggests that Dicer is not involved in agotron-mediated repression, which consolidates agotrons as a new Dicer-independent class of short regulatory RNAs. This also explains why the vast majority of agotrons (that is, introns with 5′-end aligned long HITS-CLIP reads) are not found in small RNA sequencing libraries and therefore are excluded from the miRBase. miR-151a has an almost unbiased strand selection between the 5*p* and 3*p* arm. Accordingly, in WT HEK293T cells, both the 5*p* and 3*p* reporters are efficiently repressed (Fig. 4d). In Dicer-null cells (Dicer 2-20), the 3*p* reporter is practically unaffected by miR-151a co-expression, whereas the 5*p* reporter remains suppressed albeit at lower efficiency (Fig. 4d). This suggests that the ‘undiced' pre-miR-151a still has the capacity to function as an agotron-like species, but the target reservoir is restricted to the 5*p* arm. Therefore, the miRNA maturation by Dicer cleavage could in part be required for the liberation and activation of the 3*p* arm, which in the pre-miRNA (non-diced) context is completely inert.

### Characterization of agotrons

**Figure 5:**
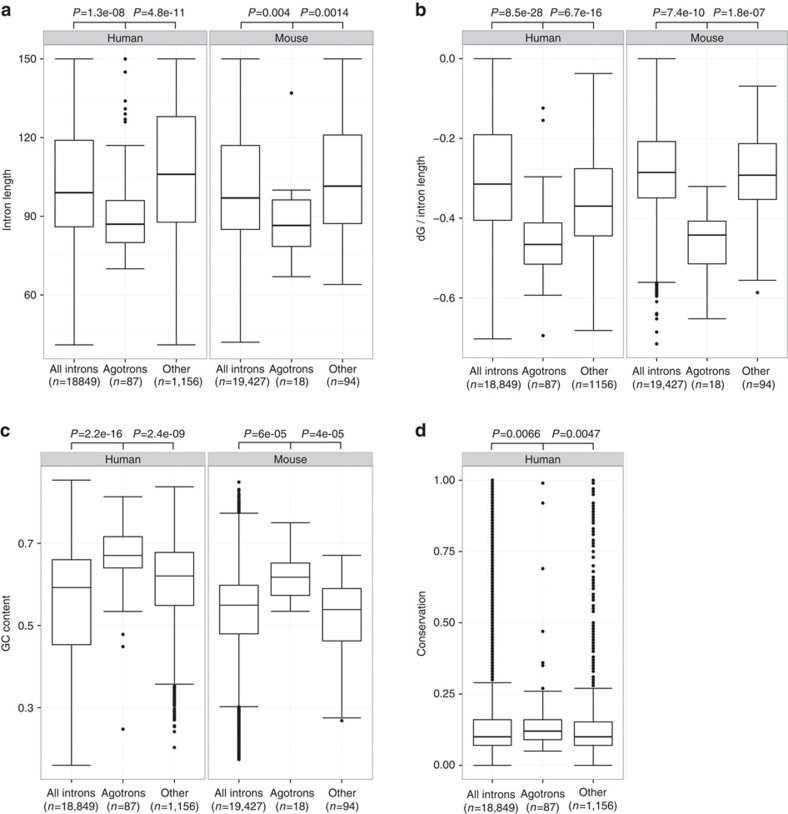
Agotrons are highly stable and GC-rich introns. Boxplots of human and murine agotrons analysed by length (**a**), free energy normalized to intron length (dG/intron length) (**b**) and GC content (**c**). Agotrons were compared with other expressed introns (at least 20 reads in two datasets) and all annotated introns. (**d**) Conservation of human agotrons compared with other expressed introns or all annotated introns. Conservation of each intron is based on the mean phastcons 46way placental conservation obtained from the UCSC genome browser.

Defining agotrons as short introns <150 nts with long (≥30 nt), 5′-end aligned (≥70%) Ago2 HITS-CLIP reads we combined all the data sets analysed (see Supplementary Data 1), to compile a more comprehensive list of putative agotrons. For both human and mouse, we selected all the introns that were expressed (≥20 reads) in at least two data sets. These introns were classified as agotrons if at least half and a minimum of two HITS-CLIP data sets adhered to the above definition. This resulted in 87 and 18 agotrons in human and mouse, respectively (Supplementary Data 2). As a class, agotrons exhibit a more restricted length distribution compared with all short introns and other expressed non-agotron introns (Fig. 5a). Moreover, for both human and mouse, the putative agotrons are significantly more structured measured by predicted free energy and also significantly more GC-rich, compared with other introns (Fig. 5b,c). Finally, based on the human set, agotrons are significantly more conserved (Fig. 5d). Even though the abundance of agotrons is generally low compared with highly expressed miRNAs, the expression levels of agotrons are very similar to mirtrons based on HITS-CLIP read per million (RPM) (Supplementary Fig. 8a,b), which probably reflect the shared biogenesis pathway. Collectively, this indicates that agotrons represent a defined family of introns that, as a class, is preserved in evolution and therefore constitutes a hitherto overlooked family of short non-coding RNA with regulatory potential.

## Discussion

Here we have characterized a novel class of short regulatory RNA, the agotrons. Unlike known Ago-associated RNA species, agotrons escape the conventional biogenesis pathway entirely and associates with Ago proteins as an unprocessed, full-length intron. We believe that agotrons are debranched introns based on the following features: (1) no branch-point traversing reads in the HITS-CLIP are detected. (2) Ago2 has been shown to associate with free 5′-ends^21^ and (3) the northern blotting analyses of agotrons adhere with full-length linear and not lariat RNA migration. The discrepancy between the observed median read length in HITS-CLIP data and northern blot migration of agotrons is probably due to the sequence length of the libraries and the RNAse digestion used in HITS-CLIP to accurately footprint the Ago-associated region.

Agotron targeting is governed by seed interactions similar to miRNAs; however, the potency is somewhat limited. The modest repression of seed-mediated targeting could be explained by the very stable secondary structure of the *Pkd1* agotron hairpin that must dissociate to allow the agotron-target interaction. This probably provides agotrons with a much more limited target repertoire compared with miRNAs and are possibly less prone to unwanted off-targeting, which potentially could be exploited in agotron-inspired RNA interference. The optimal sequence- and structural requirements for agotron behaviour is currently unknown and remains to be further explored. We observe that the *Pkd1* agotron acts independently of Dicer. In fact, in Dicer-null cells the agotron functions more effectively and without the co-expression of Ago2. This indicates that the elimination of Dicer-dependent Ago2 substrates results in an increased pool of free Ago2 and thus a more efficient Ago2 association and elevated effect on Dicer-independent substrates, such as agotrons.

Even though agotron-mediated target gene repression can be demonstrated in cell lines, the biological relevance of agotrons could also be a surveillance mechanism to avoid unwanted RNA associating with the free Ago proteins. This would increase the specificity and fidelity of Ago-loading to *bona fide* substrates and explain why agotrons are hardly detectable without an excess of Ago proteins.

In humans, the conserved *PKD1* agotron is annotated as a mirtron, miR-1225, which has previously been suggested to be splicing independent^25^. In that study, expression analysis of miR-1225 is based on RT–PCR and not northern blotting, which disables efficient demarcation between long and short RNA templates. We believe that the *Pkd1* agotron exists as a full-length intron consistent with all HITS-CLIP data analysed and with our functional results.

Conclusively, we provide evidence for the existence of a new class of short (∼80–100 nt) intronic RNA that has the potential to regulate gene expression in a miRNA-like manner. These species escape conventional small RNA profiling and therefore constitute overlooked biological molecules that, similar to miRNAs, could play key roles in differentiation and disease.

## Methods

### CLIP analysis

Sequence reads from published HITS-CLIP experiments (see Supplementary Data 1) were initially adaptor trimmed and reads below 18 nts in length were discarded. Subsequently, reads were aligned while allowing for one mismatch to annotated murine (UCSC gene annotation, mm10) or human (UCSC gene annotation, hg19) introns between 40 and 150 nucleotides in length, annotated pre-miRNAs (mirbase v. 21) or small nucleolar RNAs extracted from Ensembl BioMart. Mirtrons were defined as short introns (between 40 and 150 nts) annotated in mirbase. The number of aligned reads, the median read length and, for introns, the fraction of reads aligning within one nucleotide of the 5′-end was determined. RPM was calculated for individual samples and the average RPM for each data set was used as measurement of expression. Only regions with at least 20 reads in total were included in the analysis. Free energy (Δ*G*) was determined by MultiRNAFold^26^.

### Constructs and transfections

HEK293 Flp-In T-Rex cells (Invitrogen), HEK293T and Dicer KO HEK293T cells (gifts from Bryan Cullen Lab) were maintained under standard culture conditions. Transfections were conducted using standard calcium phosphate procedures. Agotron expression vectors were constructed by PCR amplifying the agotron with flanking exons and inserting the fragment into pcDNA3 (Invitrogen) using EcoRI and XhoI. Similarly, miR-151a expression vector was constructed by PCR amplifying an ∼500-bp genomic region encompassing the annotated pre-miR-151a and inserting the fragment into pJEBB expression vector using NotI and SalI. For *Pkd1* mutants, overlapping PCR was performed with internal primers harbouring the mutation of interest and the original flanking primers. Luciferase reporter vectors were constructed by PCR amplicons (psiCheck-Pkd1 (1–47) and psiCheck-Pkd1 (48–94)) or inserting annealed oligos into psiCheck-2 vector using XhoI and SpeI. Primers used for cloning are listed in Supplementary Data 3. Ago overexpression vectors have been published previously^27^.

### Northern blotting

For PAGE, 20–30 μg total RNA was loaded on a 12% denaturing PAGE gels. RNA was transferred to Amersham hybond-N+ membranes (GE Healthcare). The membranes were hybridized with ^32^P-labelled DNA oligos (listed in Supplementary Data 3) in church buffer (0.5 M NaPO_4_, 7% SDS, 1 mM EDTA, 1% BSA pH 7.5) at 37–50 °C and washed in 2 × SSC (300 mM NaCl, 30 mM Na-citrate pH 7.0) with 0.1% SDS at 25–40 °C. The membranes were exposed on phosphorimager screens, scanned on PMI (Bio Rad) and analysed using Image Lab software (Bio Rad).

For agarose, northern blotting was performed with 10 μg RNA separated in 1.2% agarose gel. Subsequent hybridization and wash was carried out as described above. Uncropped images of all blots in the main text are shown in Supplementary Fig. 9.

### Ago immunoprecipitation

Myc-tagged Ago2 was co-transfected with either pcDNA3-Pkd1 or an empty vector in p10 dishes. Forty-eight hours post transfection, cells were lysed in 150 mM KCl, 25 mM Tris-HCl pH 7.4, 5 mM EDTA, 0.5% Triton X-100, 5 mM dithiothreitol supplemented with Ribolock RNase Inhibitor (Thermo Scientific) and proteinase inhibitor cocktail (Roche)^28^. Ten per cent was kept as input. The remaining lysate was mixed with 2.5 μg myc-antibody (Abcam, ab9106) coupled to Dynabeads Protein A/G (Novex) and left rotating for 4 h at 4 °C. Beads were subsequently washed five times in lysis buffer and the RNA was extracted using TRIzol reagent (Invitrogen). Half the input and IP were used for northern blotting analysis.

### Fluorescence *in-situ* hybridization/immunofluorescence

HEK293 Flp-In T-Rex cells (Invitrogen) were seeded on glass coverslips in 12-well plates and transfected with Ago2 and *Pkd1* expression plasmids using calcium phosphate. Forty-eight hours after transfection, cells were washed in PBS and fixed in 4% paraformaldehyde for 15 min and permeabilized overnight in 70% ethanol. Cells were then washed twice in PBSM (PBS containing 5 mM MgCl_2_). Subsequently, cells were blocked with 3% BSA in PBS for 1 h followed by incubation with a 1:1,000 dilution of primary antibody (human Dcp1a^22^) in 3% BSA/PBSM supplemented with RiboLock RNase Inhibitor (Thermo Scientific) at 37 °C. After washing three times in PBSM, cells were incubated with 1:1,000 diluted secondary antibody (Alexa Fluor 488, A21206, Invitrogen). After 3 × washes, cells were incubated at 37 °C in a solution containing 30% UltraPure formamide (Invitrogen), 2 × SSC, 0.25 mg ml^−1^ *Escherichia coli* transfer RNA, 0.25 mg ml^−1^ UltraPure salmon sperm DNA (Invitrogen), 2.5 mg ml^−1^ BSA (Roche) and 0.5 ng ml^−1^ *Pkd1* probes (Supplementary Data 3) fluorescently labelled with cy5 using the Cy5 Mono-Reactive Dye Pack (GE Healthcare). After 3 h, cells were washed twice for 20 min at 37 °C in 30% formamide and 2 × SSC, followed by four 5-min washes in PBS (the penultimate wash containing 4′,6-diamidino-2-phenylindole) and an additional brief wash in nuclease-free water. Cells were mounted in ProLong Gold (Invitrogen) and left overnight at room temperature.

### Luciferase reporter assay

HEK293 cells seeded in 12-well plates were transfected with 0.4 μg pcDNA3/pcDNA3-Pkd1, 0.4 μg pcDNA3-Ago2 and 0.1 μg psiCheck vector, and harvested after 48 h using the Dual-Luciferase Reporter Assay kit (Promega). Luminescence was measured on a BMG FLUOstar luminometer (BMG labtech).

### RNA extraction and RT–PCR

Total RNA was extracted from cells in culture using TRIzol reagent (Life Technologies) according to standard procedures. Complementary DNA for RT–PCR expression analyses was synthesized from 1 μg total RNA by MLV-RT (Invitrogen) according to the supplied protocol using random hexamer primer. Twenty-five cycles of PCR was performed with recombinant taq DNA polymerase (Invitrogen) according to manufacturers' procedures using primers listed in Supplementary Data 3. PCR amplicons were separated by 1% agarose gel electrophoresis with SYBR safe (Life Technologies) and visualized using Gel Doc XR+ (BioRad).

### RNA deep sequencing

RNA sequencing libraries were prepared with RiboZero rRNA Removal Kit (Epicentre) followed by ScriptSeq v2 RNA-Seq Library Preparation Kit, adhering to the manufacturer's protocols. Paired-end sequencing was performed at Beijing Genomics Institute. Mapping of reads and quantification of transcripts were done using Tophat and Cufflinks using gene annotation from iGenome (from 2012, hg19).

## Additional information

**Accession codes:** Raw sequencing data has been deposited at the European Nucleotide Archive (accession PRJEB10234).

**How to cite this article:** Hansen, T. B. *et al*. Argonaute-associated short introns are a novel class of gene regulators. *Nat. Commun.* 7:11538 doi: 10.1038/ncomms11538 (2016).

## Supplementary Material

Supplementary Information Supplementary Figures 1-9

Supplementary Data 1 HITS-CLIP datasets

Supplementary Data 2 Annotated agotrons

Supplementary Data 3 Primers and Probes
