# Supplementary material for: Argonaute-associated short introns are a novel class of gene regulators
Source: Nat Commun. 2016 May 13;7:11538. doi: 10.1038/ncomms11538 (PMC4869172; doi:10.1038/ncomms11538)
Supplement: Supplementary Information — Supplementary Figures 1-9 [file ncomms11538-s1.pdf]

Supplementary Figure 1:

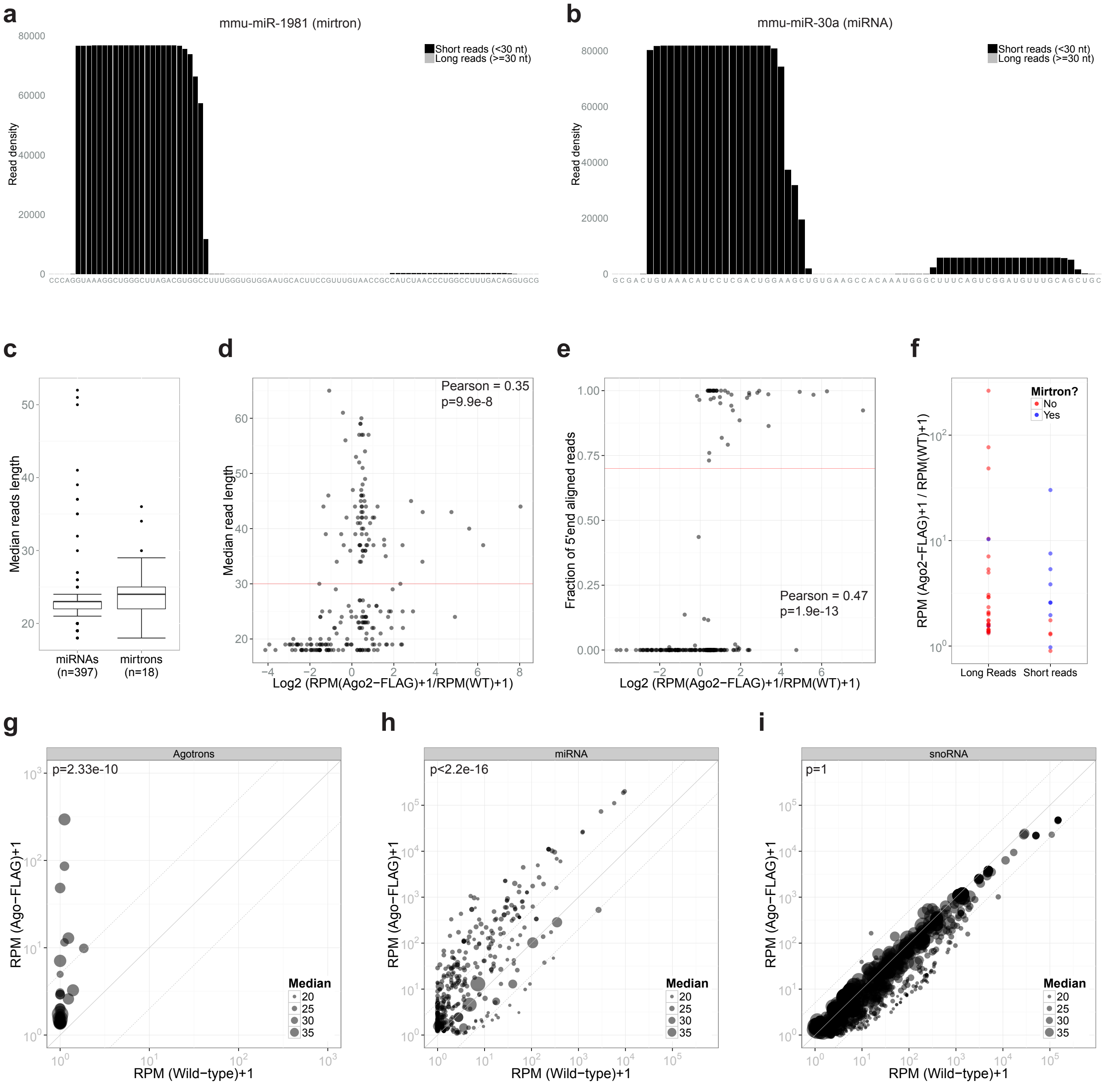

Supplementary Figure 1: Analysis of HITS-CLIP data from Tan *et al*, 2013. a-b) Read density plots (as in Fig. 1a-c) of mirtron, mmu-miR-1981 (chr1:184822402-184822493(-), mm10) (a), and miRNA, mmu-miR-30a (chr1:23272269-23272339(+), mm10) (b). **c**) Boxplot of median read length for miRNAs and mirtrons obtained in HITS-CLIP. **d-e**) The median read length (d) or the fraction of 5'end aligned reads on short introns (e) as a function of the relative enrichment in FLAG-tagged Ago2 HITS-CLIP compared to WT. The cutoff used to define agotrons (30 nt and 70%, respectively) is indicated by red line. P-values are calculated using linear regression. **f**) Enrichment in Ago2-FLAG compared to WT for all the short introns with high fraction of 5' end aligned reads (>= 70%) subgrouped into long reads (median >= 30) or short reads (median <30) color-coded by miRbase annotation as indicated. **g-i**) Scatterplot of HITS-CLIP RPM obtained in Ago2-FLAG vs. WT for agotrons (g), annotated murine miRNAs (miRBase v. 20) (h) and snoRNAs (i). The size of individual points reflects the median read length of the respective locus. P-values are calculated by one-sided sign test.

Supplementary Figure 2:

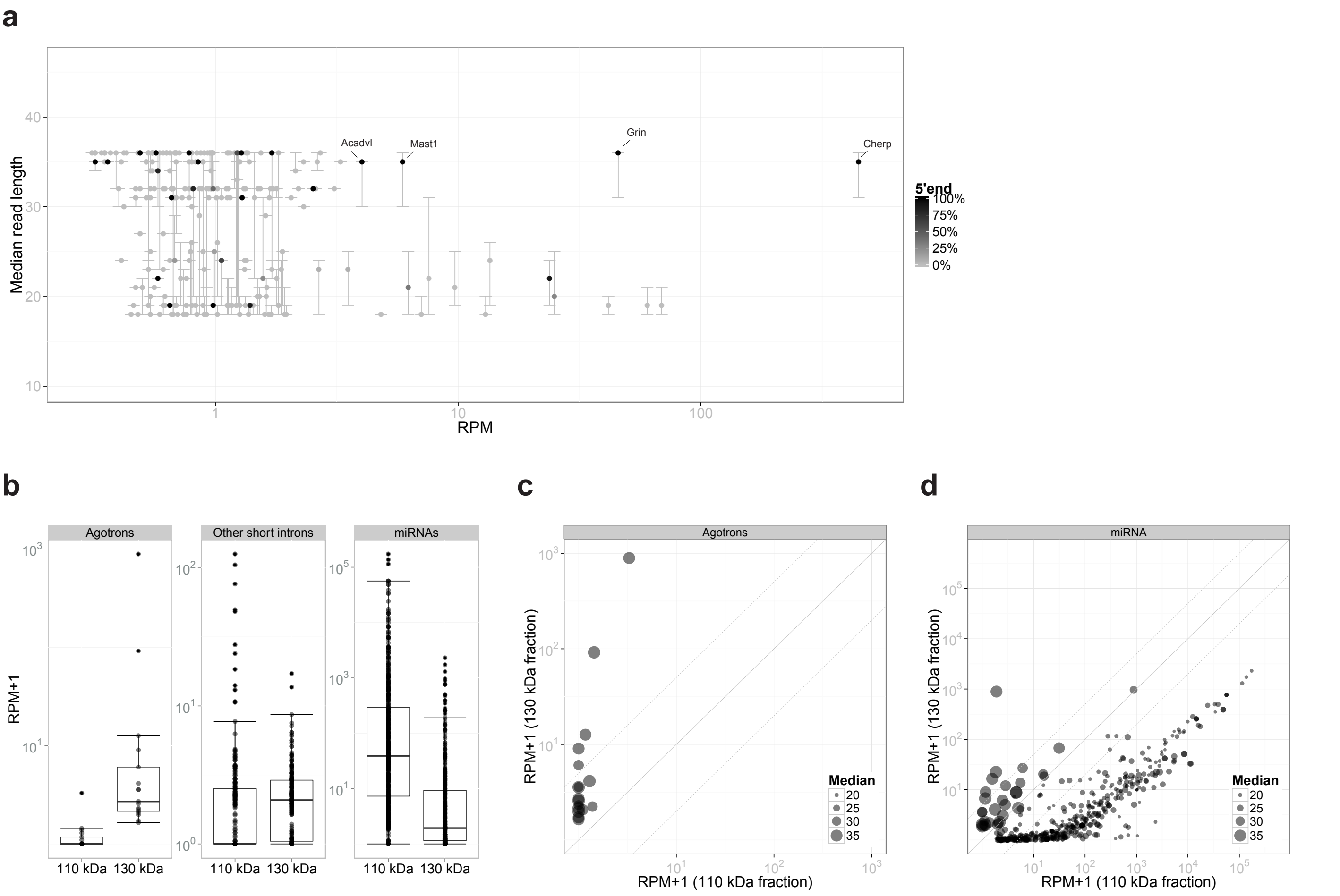

Supplementary Figure 2: Analysis of HITS-CLIP data from Chi *et al,* 2009. a) Scatterplot of median length as a function of RPM (as in Fig. 1l). **b**) Boxplots of RPM for agotrons, other introns and miRNAs obtained in the 110kDa and 130kDa fractions as indicated. **c-d**) Scatter plots of RPM in the 110kDa and 130kDa fraction on agotrons (c) or annotated miRNAs (d). Points are size-scaled according to the median read length.

Supplementary Figure 3:

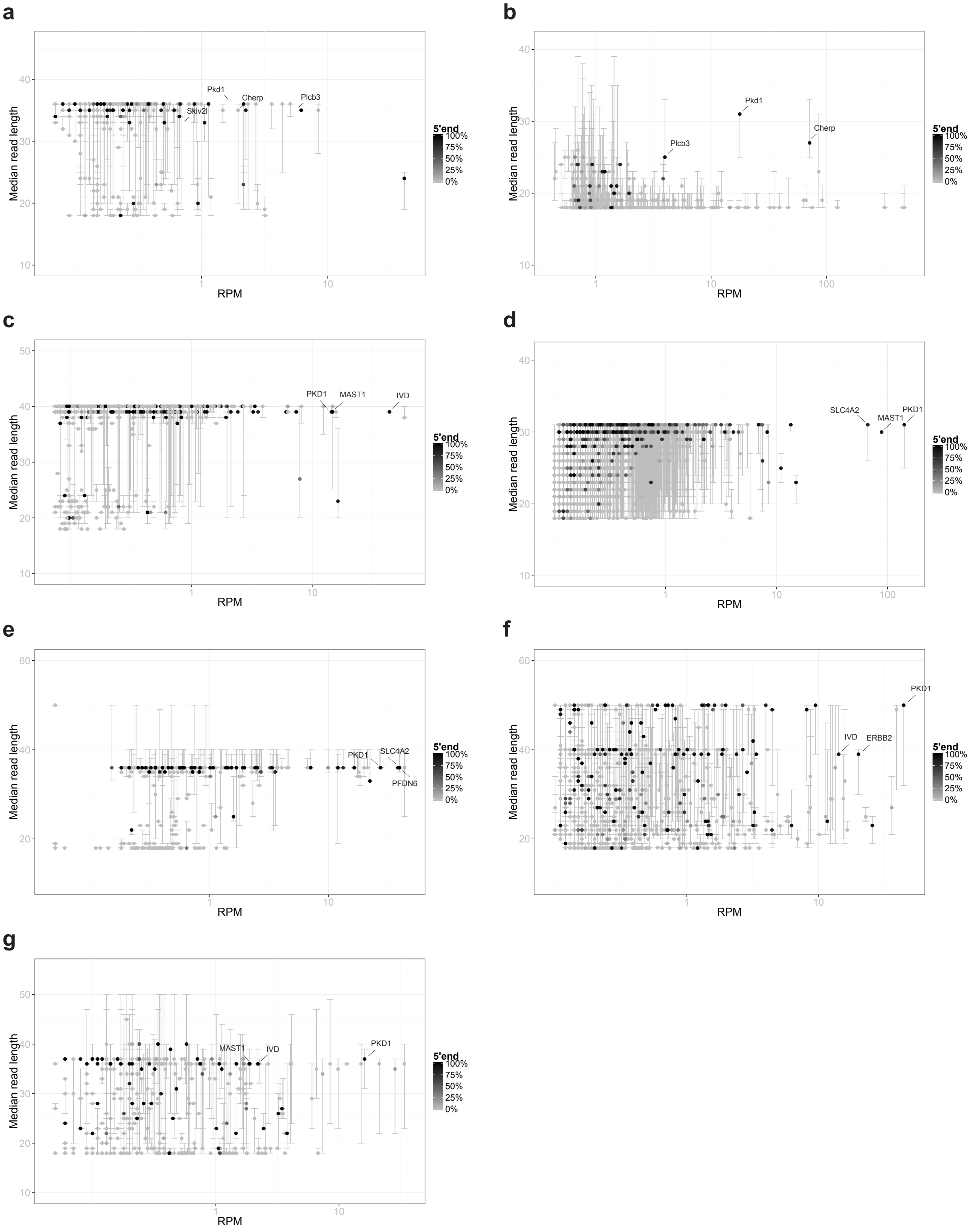

Supplementary Figure 3: Analysis of other murine and human HITS-CLIP data. a-g) Scatterplots of median length as a function of RPM (as in Fig. 1k) for published murine datasets: GSE41285 (a) and GSE25310 (b) and published human datasets: GSE41357 (c), GSE44404 (d), GSE42701 (e), GSE57855 (f), and GSE41272 (g). Color-coding reflects fraction of 5'end aligned reads. Selected agotrons are labelled. See detailed list of all datasets used in Supplementary Data 1.

Supplementary Figure 4:

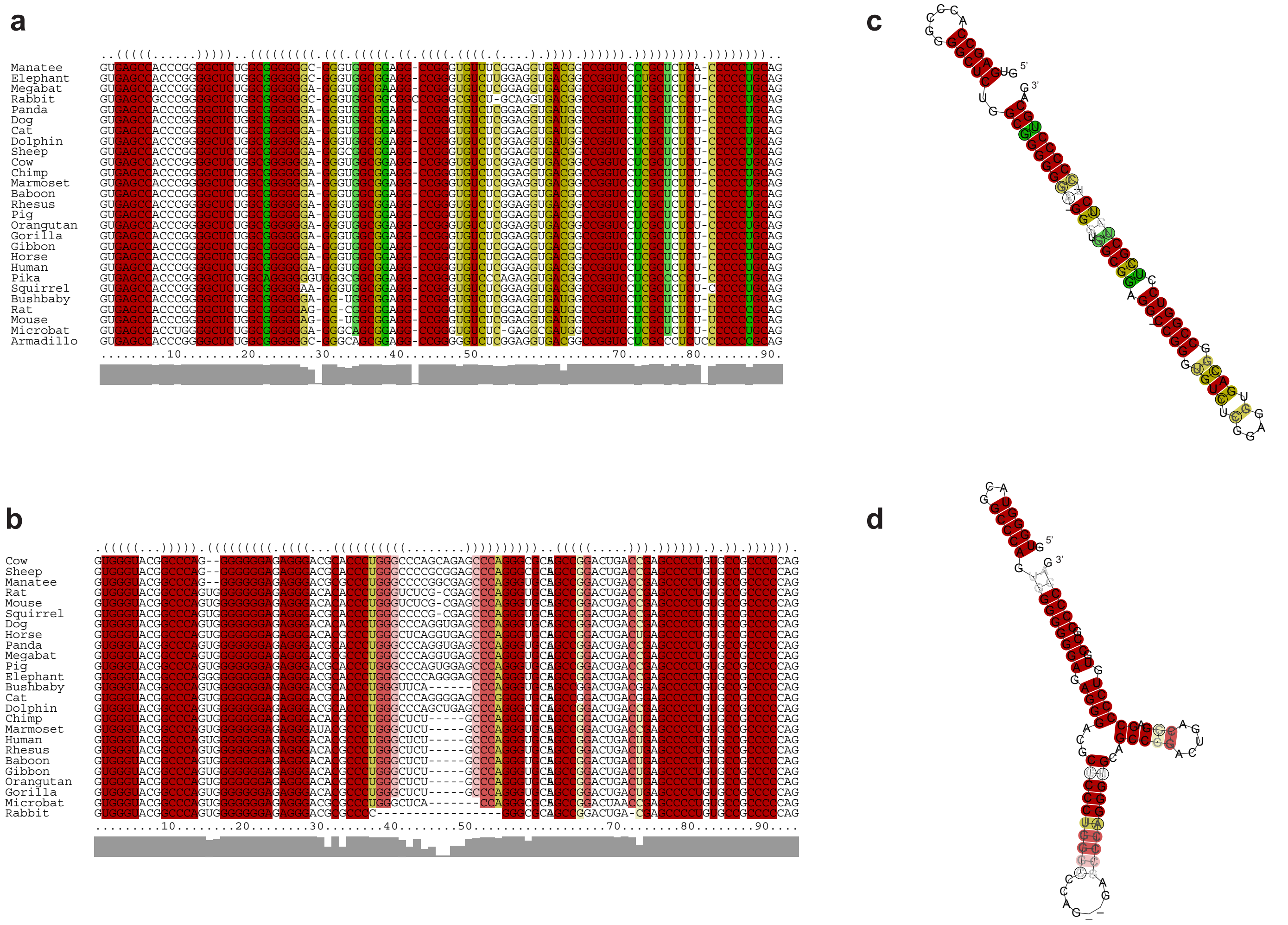

Supplementary Figure 4: Cross species conservation of agotrons. a-b) Multiple species alignment of *Mast1* (a) and *Pkd1* (b) agotrons visualized by the LocARNA-P tool. **c-d)** RNA secondary structure of *Mast1* (c) and *Pkd1* (d) predicted and visualized by LocARNA-P tool (http://rna.informatik.uni-freiburg.de/LocARNA).

Supplementary Figure 5:

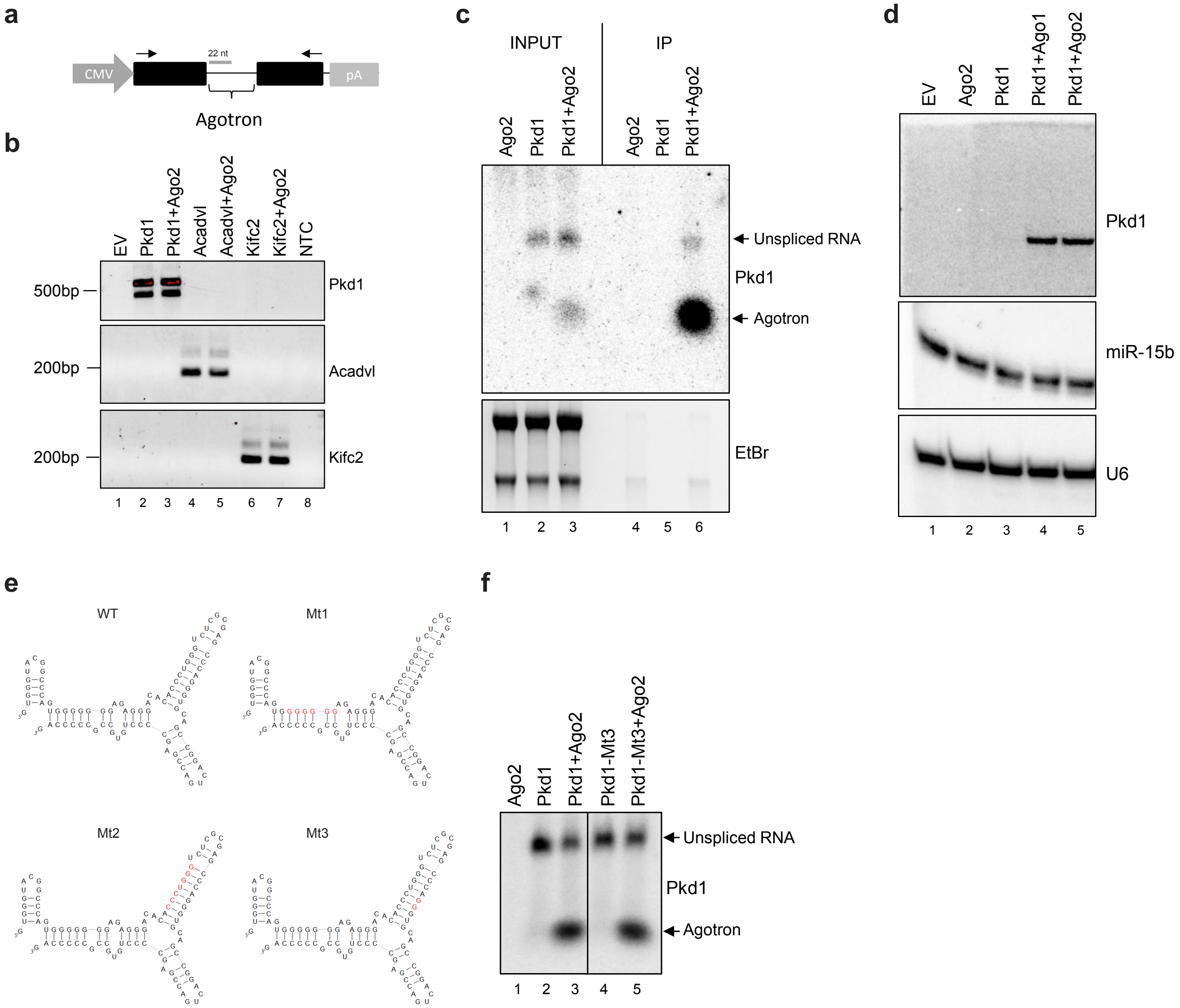

Supplementary Figure 5: Transient agotron expression. a) Schematic representation of minigene constructs with agotron and flanking exons (black bars). RT-primer (black arrows) and 22 nucleotide (nt) northern probe (grey bar) locations are depicted. CMV and pA represent promoter and terminator, respectively. **b)** RT-PCR on RNA from HEK293 cells transiently expressing Ago2 and agotron minigene as indicated. Primersets are denoted to the right. EV: Empty Vector (pcDNA3), NTC: No Template Control. **c**) Northern blotting of RNA from Ago2-IP (as in Fig. 2b). Here, the RNA is separated on a 1.2% agarose gel. Migration of unspliced RNA and agotron is marked on the right. EtBr; Ethidium bromide staining of ribosomal RNA. **d**) Transient co-expression of Ago-1 or -2 with *Pkd1* agotron. miR-15b and U6 serve as loading controls. **e**) Secondary structure prediction of *Pkd1* agotron. Mutations are highlighted in red; Mt1: GGGGGG>CCCCCC, Mt2: CCCUGGG>GGGUCCC, Mt3: GG>CC. **f**) Northern blot (as in Fig. 2f) with wild type and Mt3 *Pkd1*. Unspliced RNA and mature agotron are marked to the right.

Supplementary Figure 6:

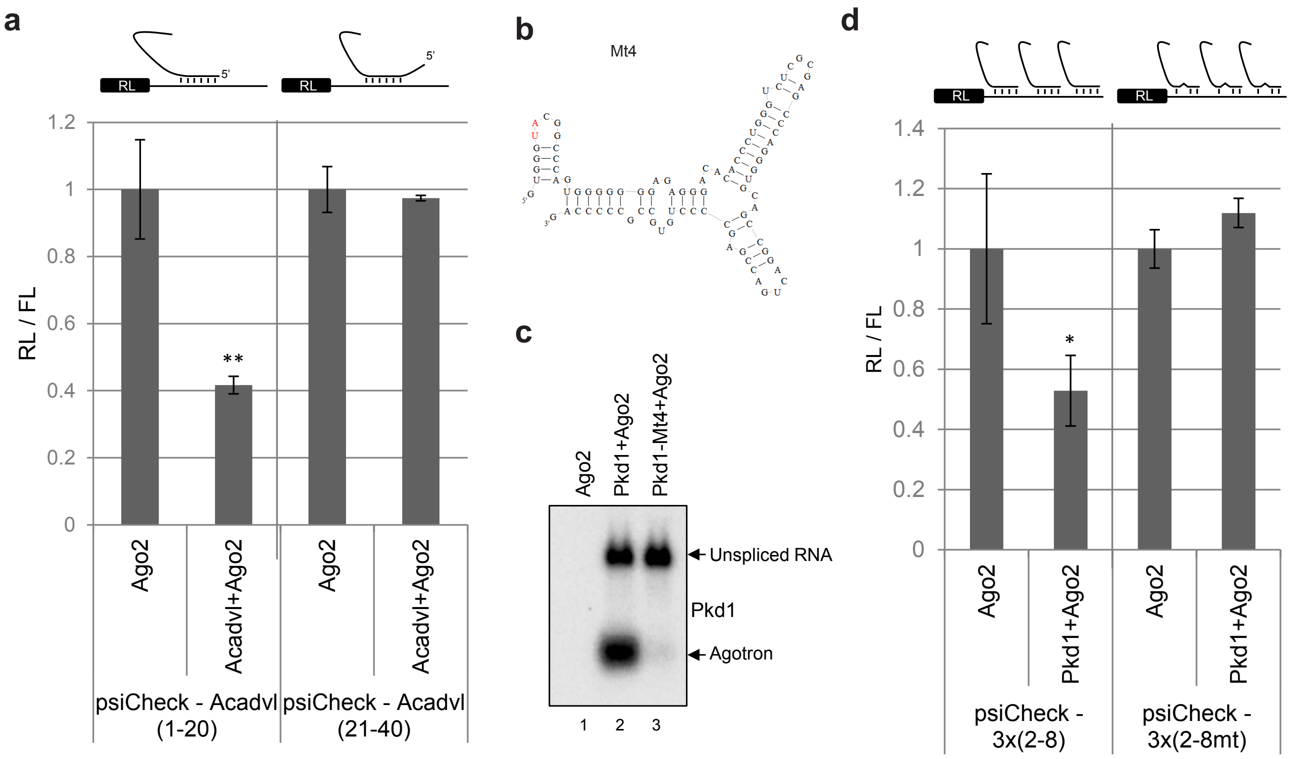

Supplementary Figure 6: Luciferase reporter assays. a) Luciferase reporter assays using psiCheck2 vectors with either a perfect target site for the indicated *Acadvl* subregions: 1-20, or 21-40 inserted in *Renilla* (RL) 3'UTR as schematically depicted. The luciferase reporters were co-expressed with either Ago2 alone (Ago2) or together with *Acadvl* (Acadvl+Ago2) and relative luminescence was determined by the *Renilla:Firefly* (RL / FL) ratio. Values were normalized to Ago2 co-expression. **b)** Secondary structure prediction of *Pkd1* agotron with seed-mutation (Mt4) highlighted in red (UA>AU). **c)** Northern blot (as in Fig. 2f) with wild type and Mt4 *Pkd1*. Unspliced RNA and mature agotron are marked to the right. **d)** Luciferase reporter assays using psiCheck2 vectors with seed-matches for wild-type and mutated *Pkd1* (3x(2-8) a 3x(2-8mt)) inserted in *Renilla* (RL) 3'UTR as schematically depicted. The luciferase reporters were co-expressed with either Ago2 alone (Ago2) or together with wildtype *Pkd1* (Pkd1+Ago2) and relative luminescence was determined by the *Renilla:Firefly* (RL / FL) ratio. Values were normalized to Ago2 co-expression. * $p < 0.05$; ** $P < 0.01$, two-tailed T-Test.

Supplementary Figure 7:

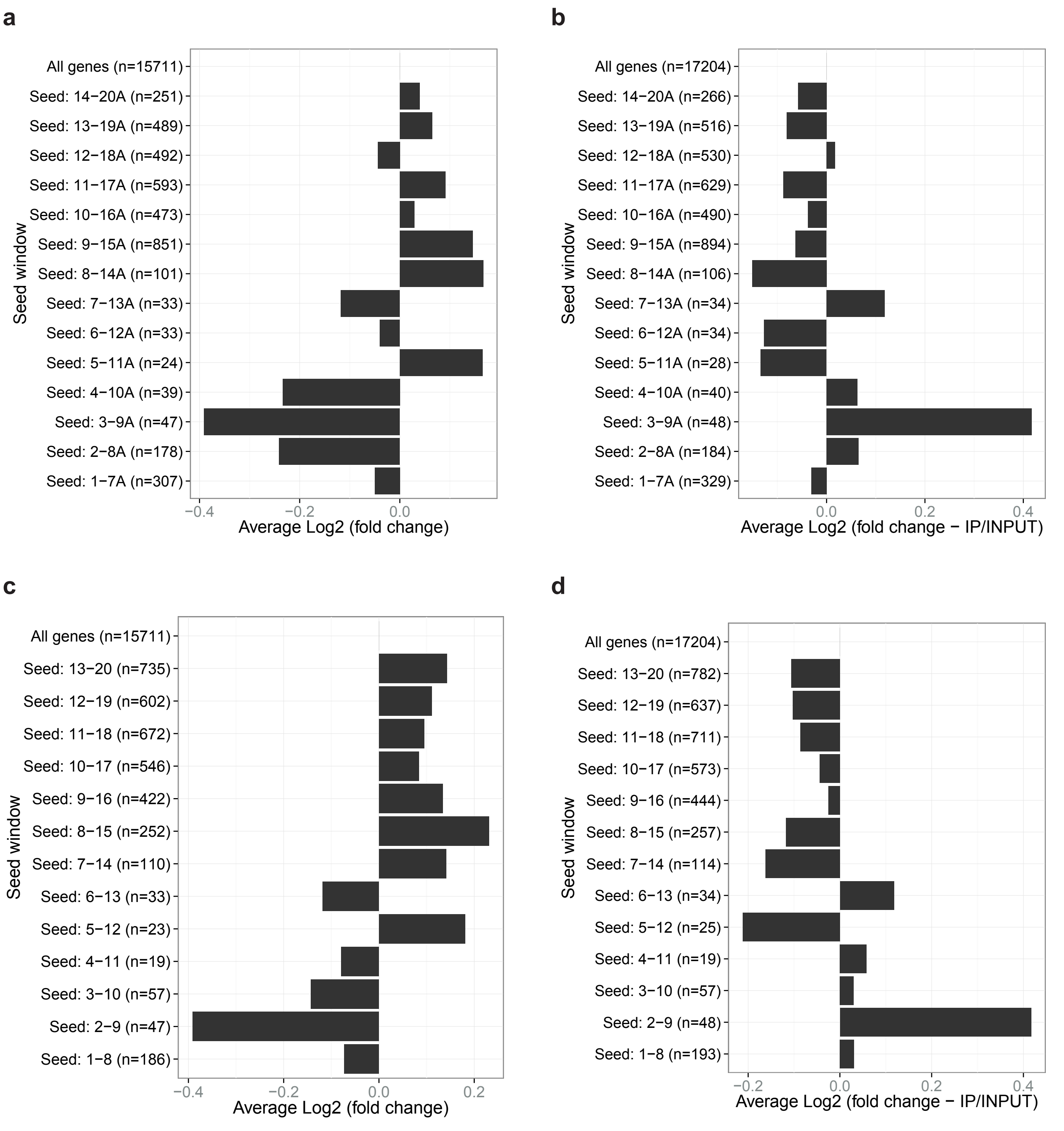

Supplementary Figure 7: Sliding seed window analysis. a-d) Average log2 fold change of targets in input (a and c) and in Ago2 IP (b and d) with sliding 7 nt seed matches with flanking A or (a and b) or sliding 8 nt seed matches (c and d).

Supplementary Figure 8:

a

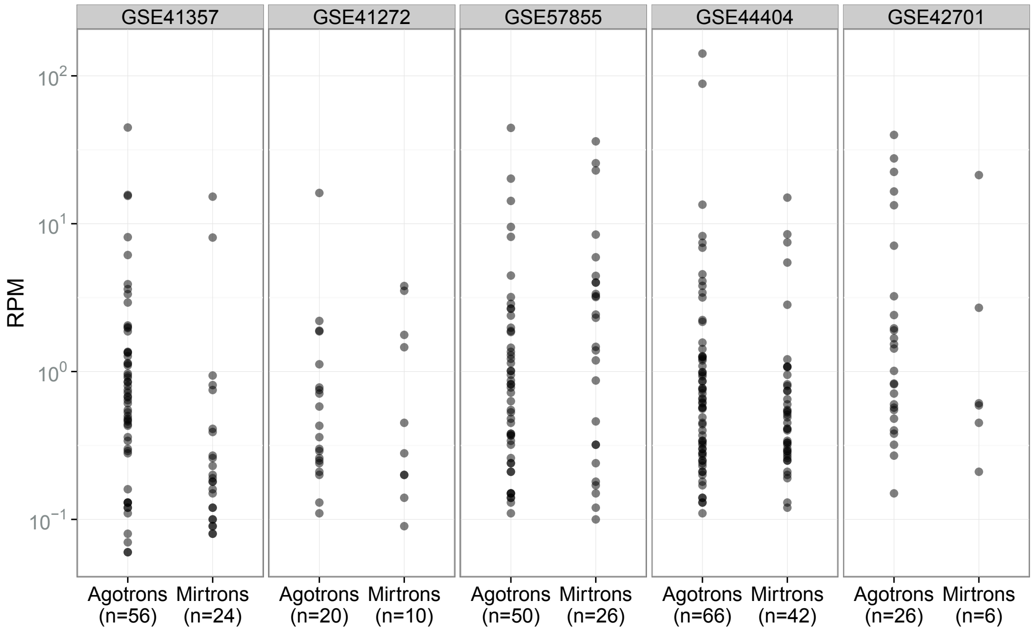

b

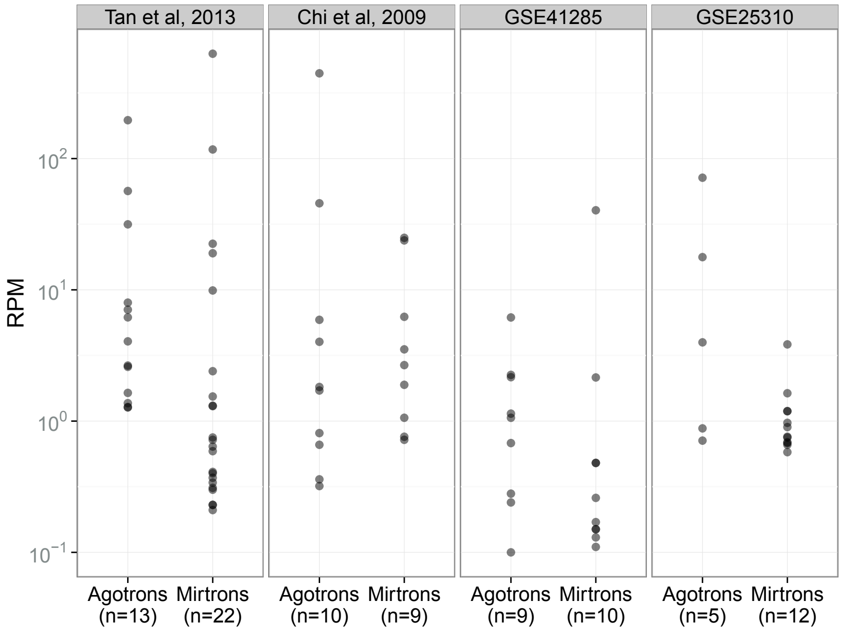

Supplementary Figure 8: Expression levels of agotrons and mirtrons. a-b) RPM values of all expressed agotrons and mirtrons based on HITS-CLIP datasets as indicated in humans (a) and mouse (b). Re-classified mirtrons are only depicted as agotrons.

Supplementary Figure 9:

From Figure 2a

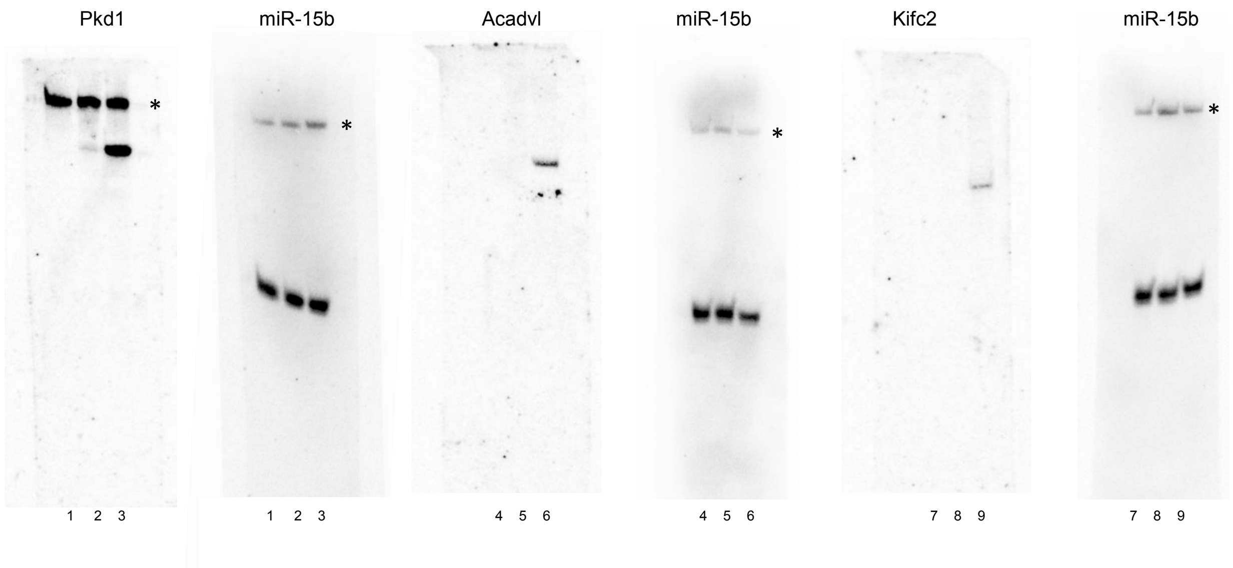

From Figure 2b

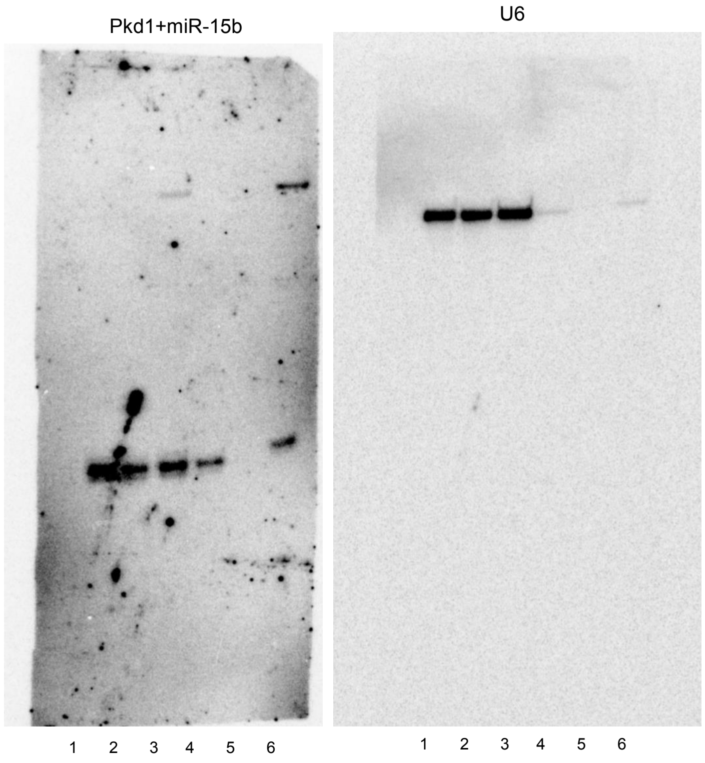

Supplementary Figure 9: Uncropped blots from main figures. Lanes, probes and corresponding main figures as specified. Asterisks denote unspecific bands.

Supplementary Figure 9 (continued):

From Figure 2d

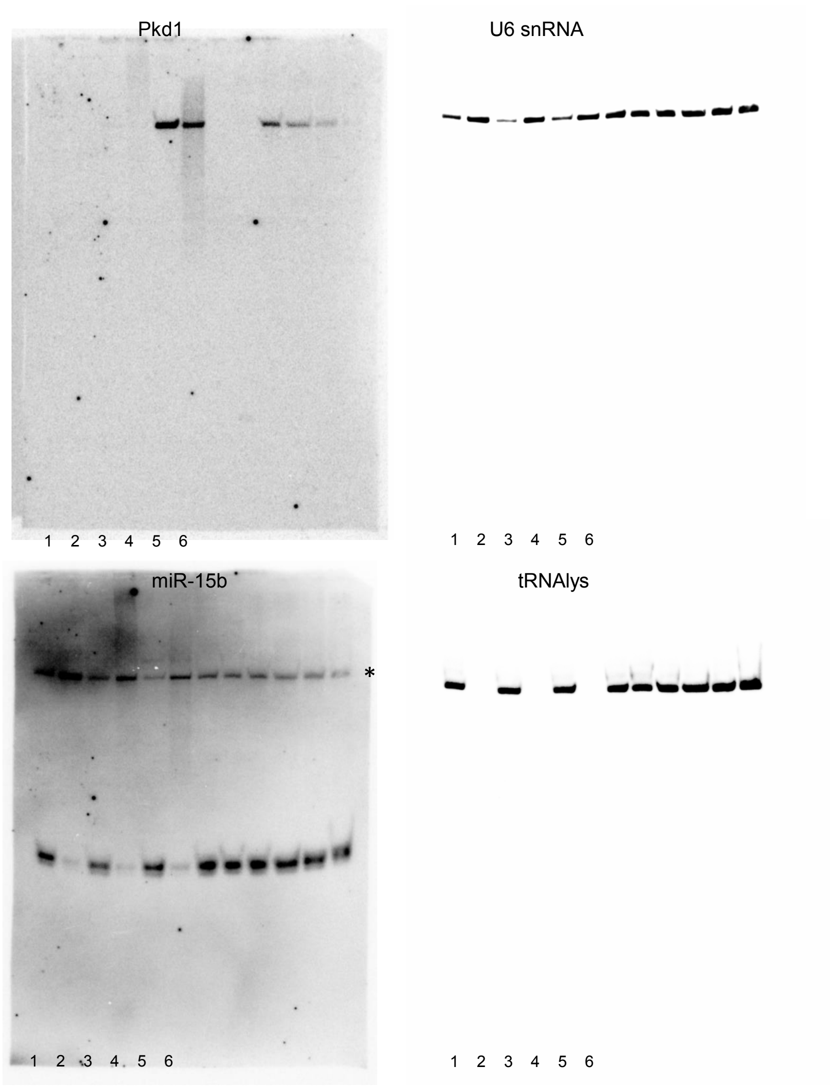

From Figure 2e

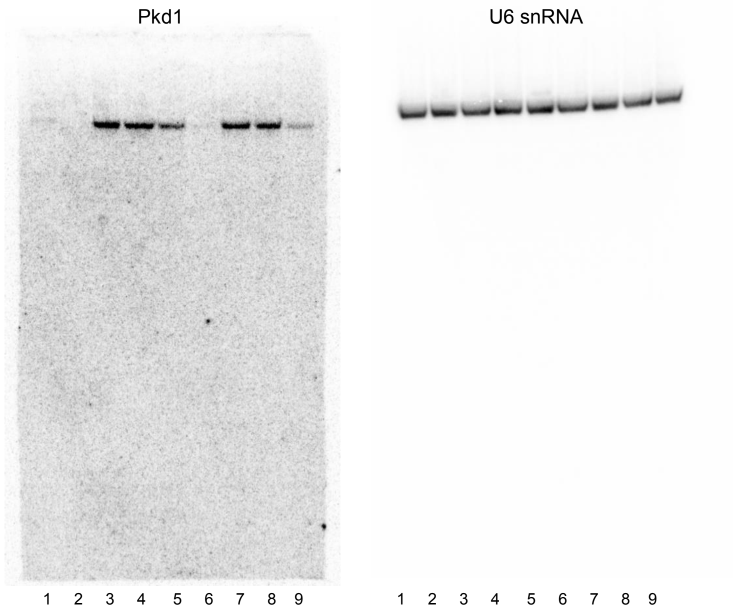

Supplementary Figure 9 (continued):

From Figure 2f

Pkd1

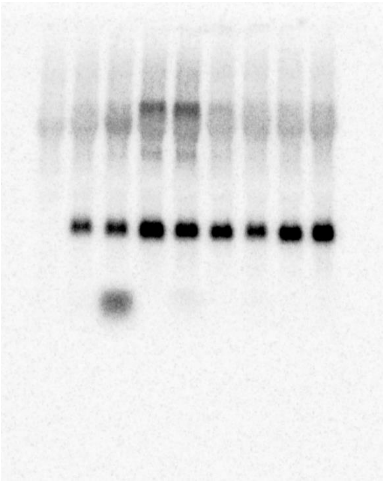

1 2 3 4 5 6 7 8 9

From Figure 4b

Pkd1 miR-151-5p U6

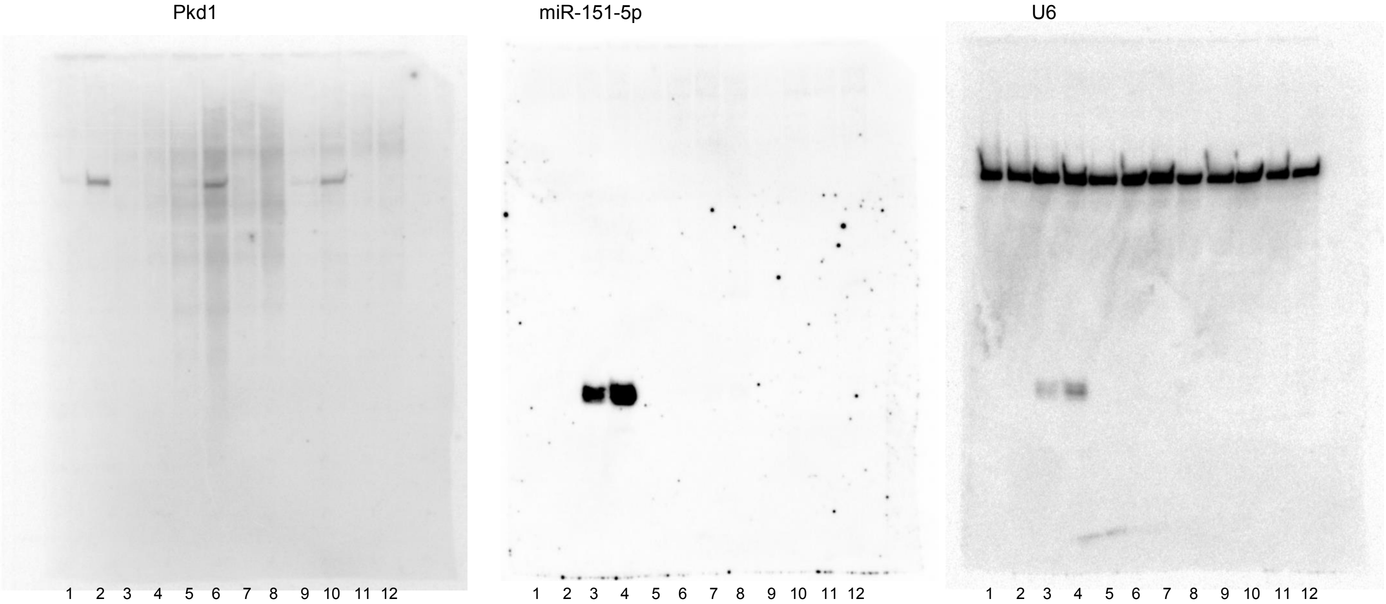

1 2 3 4 5 6 7 8 9 10 11 12 1 2 3 4 5 6 7 8 9 10 11 12 1 2 3 4 5 6 7 8 9 10 11 12
